# *Salmonella-*induced inhibition of β3-adrenoceptor expression in tumors and reduces tumor metastasis

**DOI:** 10.7150/jca.92024

**Published:** 2024-01-01

**Authors:** Li-Hsien Wu, Yu-Ting Huang, Chung-Yu Lin, Che-Hsin Lee

**Affiliations:** 1 Department of Biological Sciences, National Sun Yat-sen University, Kaohsiung 80424, Taiwan.; 2 School of Medicine, College of Medicine, National Sun Yat-sen University, Kaohsiung 80424, Taiwan.; 3 Department of Medical Research, China Medical University Hospital, China Medical University, Taichung, Taiwan.; 4 International PhD Program for Science, National Sun Yat-sen University, Kaohsiung 80424, Taiwan.; 5 Aerosol Science Research Center, National Sun Yat-sen University, Kaohsiung 80424, Taiwan.

**Keywords:** *Salmonella*, β3-adrenoceptor, tumor-targeting, migration, metastasis

## Abstract

The β3-adrenoceptor is a protein responsible for regulating the body's response to the neurotransmitter adrenaline and the hormone norepinephrine. It is critical in various physiological processes, including metabolism, thermogenesis, and cardiovascular function. Recently, researchers have discovered that the β3-adrenoceptor is also implicated in tumor progression and metastasis. Infections caused by *Salmonella* can lead to gastroenteritis; however, intriguingly, *Salmonella* is associated with tumor inhibition. In this study, *Salmonella* treatment resulted in the downregulation of β3-adrenoceptor expression and a decrease in the phosphorylation of the Protein Kinase-B (AKT)/Mammalian Target of Rapamycin (mTOR) pathway, as observed through immunoblotting in a dose-dependent manner. Notably, *Salmonella* treatment significantly reduced tumor cell migration, as demonstrated by wound healing and Transwell assays. Moreover, tumor-bearing mice that received *Salmonella*-pre-treated tumor cells exhibited improved survival rates compared to those injected with tumor cells without prior *Salmonella* treatment. The observed anti-metastatic effect in this study suggests that *Salmonella* treatment could hold promise as a potential therapeutic approach to combat tumor metastasis. Further research is warranted to explore its full therapeutic potential.

## 1. Introduction

The adrenergic β-3 receptor (β3-adrenoceptor) is a β-adrenergic receptor member of the adrenergic receptor group of G-protein-coupled receptors 1. It is encoded by the human gene *ADRB3*. The β3-adrenoceptor plays an essential role in energy processes. It is mainly found in adipocytes and is stimulated by norepinephrine and epinephrine 2. The actions of the β3-adrenoceptor include enhancement of lipolysis in adipose tissue and thermogenesis in skeletal muscle 3. The β3-adrenoceptor is involved in obesity and metabolic disorders, but its connection to tumor therapy remains unclear. Recently, the β3-adrenoceptor was found to it overexpressed in tumor cells, and the β3-adrenoceptor plays an essential role in tumor growth, invasion, or metastasis 4. This finding could potentially have implications for cancer research and treatment strategies.

Tumor metastasis is a complex process involving multiple genetic and cellular changes. The development of metastases is a significant concern in tumor treatment because it is responsible for most tumor-related deaths 5. When a tumor spreads to other body parts, treating it becomes more challenging, as it may not respond as well to localized therapies, such as surgery or radiation, that work on the primary tumor 6. Preventing metastasis is a critical goal in cancer treatment and management. While it's challenging to eliminate the risk of metastasis, it is worth studying to have an excellent way to reduce tumor metastasis.

*Salmonella* is a Gram-negative type of bacteria known for causing foodborne illnesses. Some studies used *Salmonella* as an anti-tumor biological macromolecule, particularly in the context of targeted tumor treatments 7. In our recent research, we made an intriguing discovery that *Salmonella* possesses the ability to suppress tumor metastasis by inhibiting heparanase, matrix metalloproteinase 9 (MMP-9), and C-X-C motif chemokine receptor 4 (CXCR4) 5, 8, 9. This finding adds to the growing body of evidence supporting the potential therapeutic applications of *Salmonella* in combating metastatic tumors 10. The presence of *Salmonella* in tumor cells can trigger intrinsic apoptotic pathways. This involves the activation of mitochondrial pathways leading to cell death. The bacteria can induce stress within the tumor microenvironment, which can lead to the activation of apoptotic signaling. Our previous research also found that *Salmonella* can induce apoptosis and autophagy in tumor cells 7, 10. Because *Salmonella* can hinder the growth of primary tumors and reduce tumor metastasis, *Salmonella* may be the most potent weapon against tumors 11. In this study, we want to elucidate the underlying mechanism of anti-tumor metastatic effects by *Salmonella*.

## 2. Materials and Methods

### 2.1. Cell lines and mice

Murine B16F10 melanoma and Lewis lung carcinoma (LL2) cells were cultured in Dulbecco's modified Eagle's medium (DMEM) supplemented with 50 μg/ml gentamicin, 2 mM L-glutamine, and 10% heat-inactivated fetal bovine serum (FBS) at 37℃ in 5% CO_2_ 12, 13. Female C57BL/6 mice aged 6 to 8 weeks were obtained from the National Laboratory Animal Center of Taiwan. The animals were maintained in a specific pathogen-free animal care facility under isothermal conditions with regular photoperiods. The experimental protocol adhered to the rules of the Animal Protection Act of Taiwan and was approved by the Laboratory Animal Care and Use Committee of the National Sun Yat-sen University.

### 2.2. Bacteria, plasmid, and reagents

A vaccine strain of *Salmonella enterica* serovar* choleraesuis* (*S.* Choleraesuis; S.C.) was obtained from Bioresources Collection and Research Center (Hsinchu, Taiwan) 14. Dr. Chiau-Yuang Tsai (Department of molecular immunology, Osaka University) kindly provided the active AKT plasmid. 4',6-diamidino-2-phenylindole (DAPI) was purchased from Sigma Aldrich (Sigma Aldrich, St. Louis, MO, USA).

### 2.3. The infection of *Salmonella*

The B16F10 and LL2 tumor cells (10^5^ cells/well) were cultured overnight. Subsequently, 0, 10^5^, 10^6^, and 10^7^ colony-forming units (cfu) of *Salmonella* were added to these cells cultured in 1 ml of antibiotic-free medium. Tumor cells were incubated with* Salmonella* for 1.5 h at 37℃. All the cells were washed, replenished with gentamicin (100 μg/ml)-containing complete medium, and maintained for 16 h 12, 14.

### 2.4. Immunoblotting and transfection

Tumor cells (B16F10 and LL2) were seeded at a density of 10^5^ cells/well in a 6-well plate. The medium was removed, and the cells were washed twice with 1 mL of PBS at room temperature. Subsequently, 200 μL of lysis buffer containing 150 mM NaCl, 0.5% NP40, 1 mM EDTA, and 150 mM Tris-HCl (pH 8) at room temperature was added to each well. The cells were allowed to lyse for 10-15 minutes, and the lysate was collected into microcentrifuge tubes after centrifugation at 12,000 rpm for 20 minutes. The supernatant was collected to determine the protein content using a bicinchoninic acid (BCA) protein assay (Pierce Biotechnology, Rockford, IL, USA).

Whole-cell protein samples (40 μg) were loaded onto 8% or 10% SDS-PAGE gels and transferred onto hybond-enhanced chemiluminescence nitrocellulose membranes (Pall Life Science, Glen Cove, NY, USA). The membranes were then blocked and incubated with primary antibodies (Table S1) at 4°C overnight, followed by incubation with secondary antibodies (rabbit anti-mouse IgG-peroxidase antibody at 1:10000 and goat anti-rabbit IgG-peroxidase antibody at 1:5000) (Sigma Aldrich) for 2 hours at room temperature. Protein-antibody complexes were detected using an enhanced chemiluminescence system (T-Pro Biotechnology, New Taipei City, Taiwan), and the signals were quantified using ImageJ software 15. Lipofectamine 2000 transfected cells with constitutively active AKT plasmids for AKT activation experiments. After post-transfection or treatment, the cells were treated with S.C. for 90 minutes or left untreated. Subsequently, the cell lysates were harvested and analyzed as described above.

### 2.5. Wound healing and Transwell Assays

The wound healing assay followed the manufacturer's instructions (IBIDI, Martinsried, Germany). For this, cells were seeded into the Culture-Insert 2 Well at 3 × 10^4^ cells/well density. After cell attachment, a cell-free gap was created to visualize cell migration. The migration distance was measured 24 hours later using a microscope. The migration distances of untreated cells were used as a reference and set to 100%. These distances were compared with cells treated with S.C. (multiplicity of infection (M.O.I.) = 100). 8.0 μm Millicell® Hanging Cell Culture Inserts (MilliporeSigma, Burlington, MA, USA) were used for the Transwell assay. The Transwell inserts were placed in a 24-well plate and seeded with 2 × 10^5^ cells in the upper chamber (200 μl of cell solution per chamber). Additionally, 500 μl of FBS was added to each well. The Transwell cultures were then incubated at 37℃ with 5% CO2 for 24 hours. After incubation, each well was treated with S.C. (M.O.I. = 100) and a control treatment (M.O.I. = 0) for 90 minutes. Subsequently, all the cells were washed, and the medium was replenished with gentamicin (100 μg/ml)-containing the complete medium and further cultured for 16 hours. The cells that penetrated the membrane were fixed with formaldehyde for 3 minutes and stained with DAPI (Sigma-Aldrich) for 1 minute. Finally, the cells were observed and counted under a fluorescent microscope.

### 2.6. The metastasis of animal model

The B16F10 (10^5^) and LL2 cells (10^5^) were admixed with or without* Salmonella* (MOI = 100) for 90 min, and C57BL/6 mice were injected with *Salmonella*-treated or non-treated-cells via the tail vein on Day 0. Tumor-bearing mice were sacrificed, and lungs were removed, weighed, and histologically examined on day 20. The β3-adrenoceptor content was measured by Western blotting. In a parallel experiment, mice were monitored for survival.

### 2.7. Statistical Analysis

We determined differences between groups using an unpaired, two-tailed Student's t-test. The Kaplan-Meier survival curve and log-rank test to measure a survival analysis. A p-value less than 0.05 was considered to be statistically significant.

## 3. Results

### 3.1. The inhibition of β3-adrenoceptor expression by* Salmonella* infection

**Figure 1:**
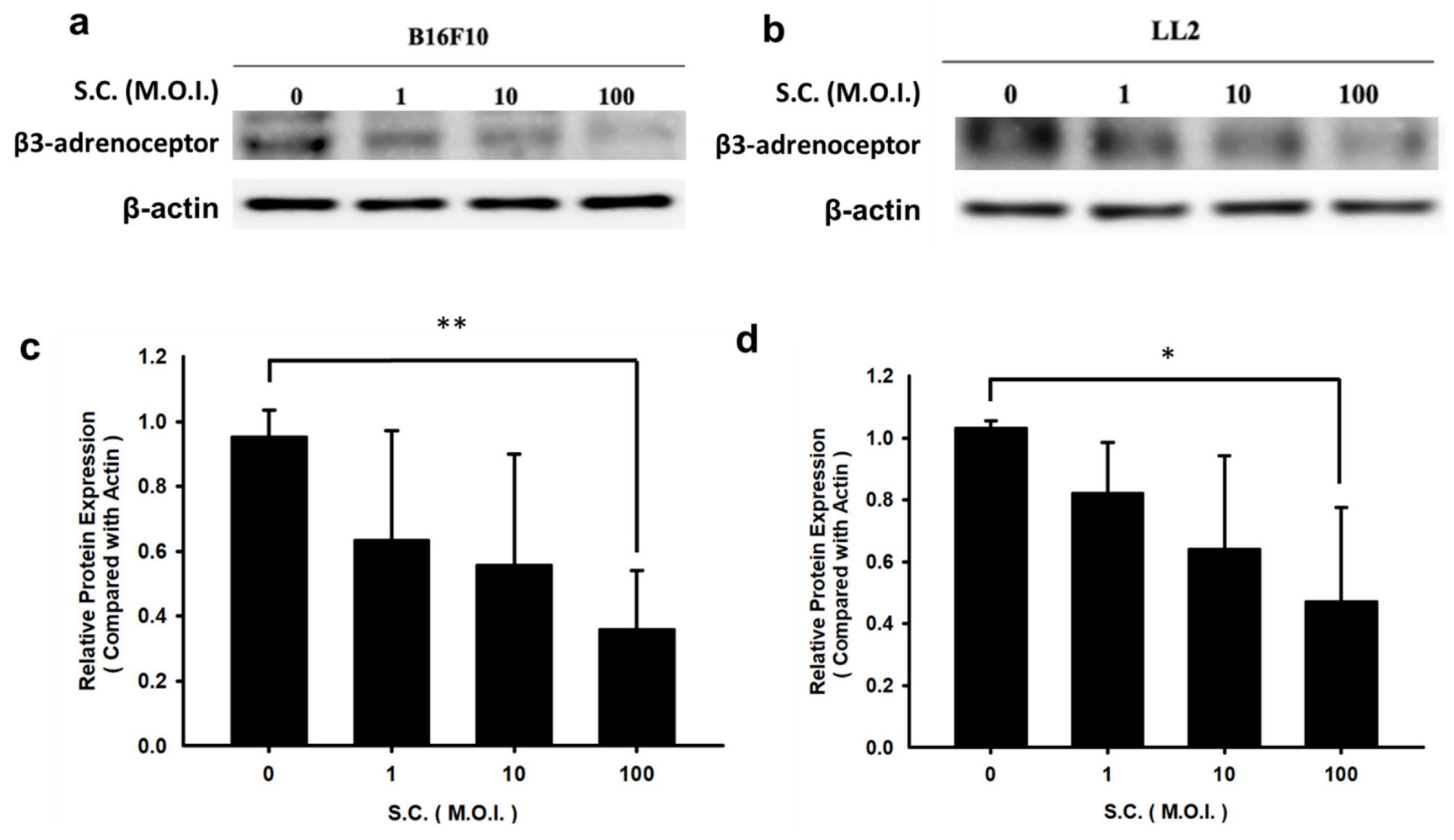
The expression levels of β3-adrenoceptor in tumor cells after *Salmonella* (S.C.) treatment. (a) The expression of β3-adrenoceptor in B16F10 and LL2 cells after *Salmonella* (S.C.) treatment. Tumor cells infected with* Salmonella* (S.C.) (multiplicity of infection (M.O.I.) = 0, 1, 10, and 100). The expression of β3-adrenoceptor was measured in B16F10 (a) and LL2 (b) cells by Western blotting analysis. The β3-adrenoceptor expression levels following *Salmonella* treatment in (c) B16F10 and (d) LL2 cells. Quantified band intensities normalized to β-actin. (n = 3, mean ± SD. * p < 0.05; ** p < 0.01).

β3-adrenoceptor is overexpressed in tumor cells 4. Due to *Salmonella*'s ability to influence the protein expression of tumor cells, we subjected the tumor cell lines to varying multiplicities of infection (M.O.I.) of *Salmonella* during our study. However, it was observed that* Salmonella* did not significantly impact cell viability, even at the highest dose (M.O.I. = 100) after 90 minutes (Data not shown). *Salmonella* reduced the expression of β3-adrenoceptor dose-dependently in B16F10 and LL2 tumor cells (Fig. 1). Based on these findings, it appears that *Salmonella* may have a regulatory effect on β3-adrenoceptor protein expression in tumor cells.

### *3.2. Salmonella* treatment resulted in a reduction of tumor cell migration

**Figure 2:**
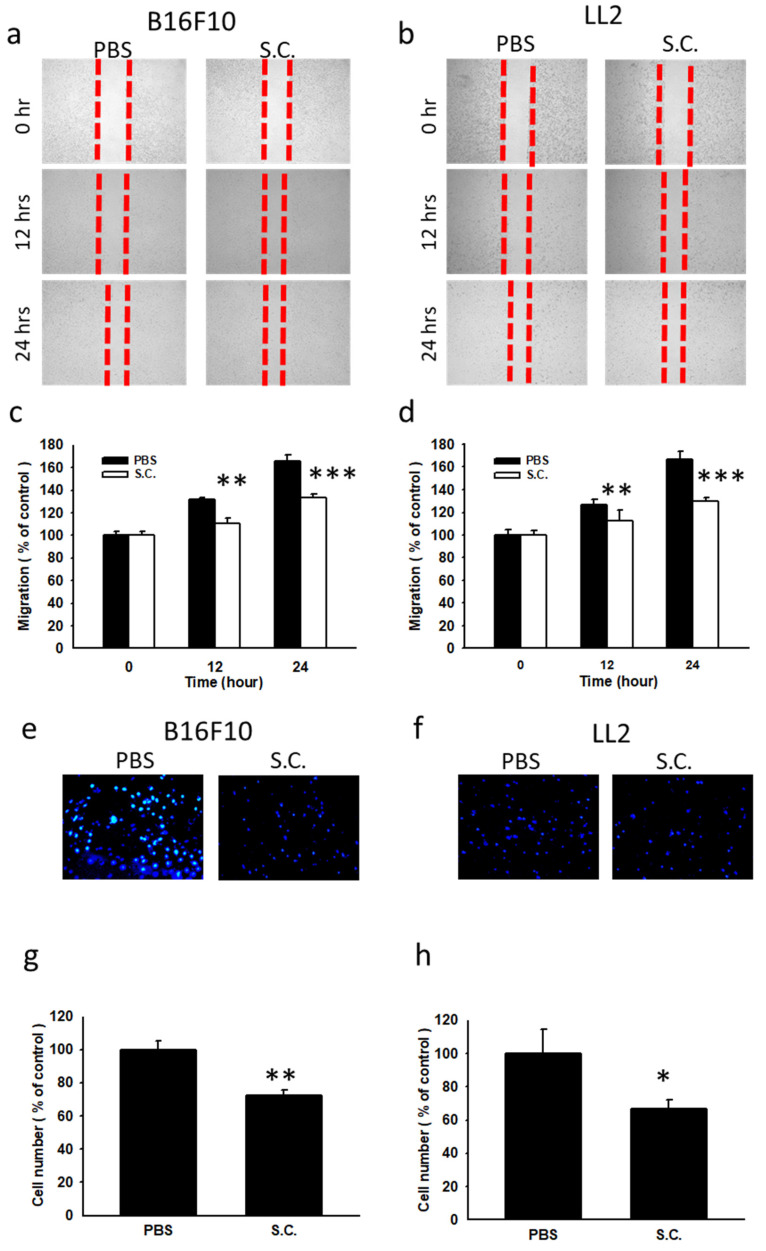
The cellular motility of B16F10 and LL2 cells after *Salmonella* (S.C.) treatment. The cells were co-cultured with *Salmonella* (MOI = 100) for 1.5 h. The motility distances of different groups of B16F10 (a) and LL2 (b) cells were measured and shown in (c, d). The B16F10 (e) and LL2 (f) cells were placed on the upper layer of Tranwell and then infected with *Salmonella* (MOI = 100) for 90 min. After 24 h, the bottom layer of cells was stained with 4',6-diamidino-2-phenylindole (DAPI) and counted under a fluorescence microscope in B16F10 (g) cells and LL2 (h) cells (n = 6, mean ± SD. * p < 0.05; ** p < 0.01; ***p < 0.001).

In this study, we investigated the impact of *Salmonella* treatment on B16F10 mouse melanoma and LL2 mouse Lewis lung carcinoma regarding their migration capabilities. To assess cell motility, a wound healing test was conducted (Fig. 2 a and b). The results from the wound healing assay revealed significant inhibition of B16F10 cell movement upon *Salmonella* treatment, compared to the control group (Fig. 2 a and c). A similar phenomenon was observed in LL2 cells infected with *Salmonella* (Fig. 2 b and d). While* Salmonella* did not affect cell proliferation during the brief infection period, the possibility of reduced cell proliferation post-*Salmonella* infection was not ruled out. Furthermore, the Transwell assay evaluated cell migration after *Salmonella* treatment (Fig. 2 e and f). The results demonstrated a significant reduction in the migration of both B16F10 and LL2 cells following treatment with *Salmonella* (Fig. 2 g and h). The number of tumor cells migrated in response to *Salmonella* treatment was notably decreased, indicating a substantial impact on the motility of the two tumor cell types. Our findings confirm that *Salmonella*-induced downregulation of β3-adrenoceptor contributed to the reduction of tumor cell metastasis *in vitro*. These results shed light on the potential therapeutic role of *Salmonella* in controlling tumor metastasis through β3-adrenoceptor regulation.

### 3.3. *Salmonella* was found to decrease β3-adrenoceptor expression via the phospho-protein kinase B (P-AKT) / phospho-mammalian target of rapamycin (P-mTOR) pathway

**Figure 3:**
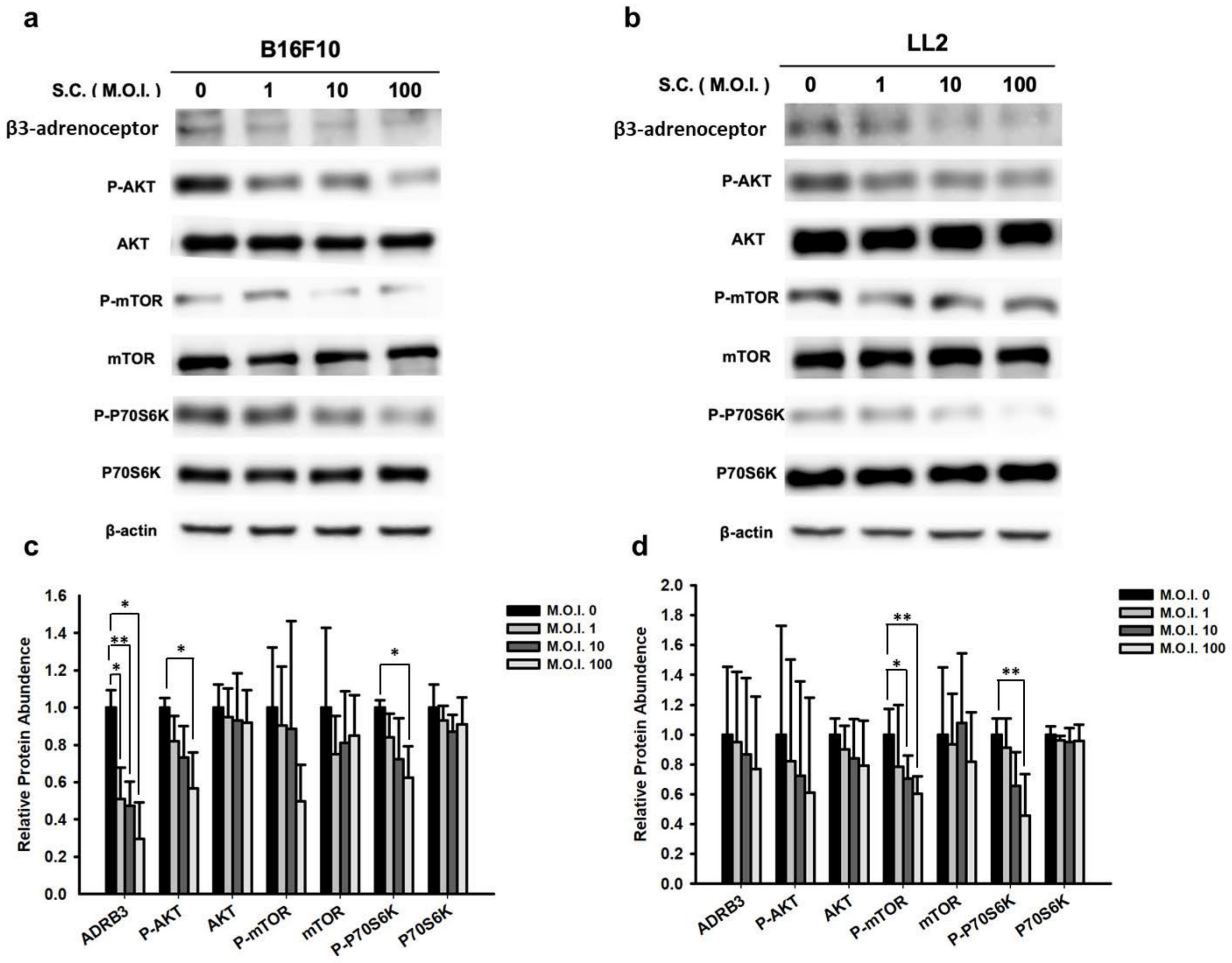
The β3-adrenoceptor expression in *Salmonella* (S.C.)-treated-B16F10 and -LL2 cells. The cells were co-cultured with *Salmonella* (MOI = 0-100) for 1.5 h. The expression of proteins in (a) B16F10 and (b) LL2 cells was measured. The immunoblotting assay was repeated three times with similar results. The AKT/mTOR/p70S6K phosphorylation expression levels following *Salmonella* treatment in (c) B16F10 and (d) LL2 cells. Quantified band intensities normalized to β-Actin. (n = 3, mean ± SD. * p < 0.05; ** p < 0.01; *** p < 0.001).

In our study, we observed a significant decrease in the protein levels of β3-adrenoceptor in *Salmonella*-treated tumor cells (Fig. 3 a and b). As the β3-adrenoceptor promotes tumor metastasis 16, we measured its expression in tumors, which was also reduced in *Salmonella*-treated tumor cells (B16F10 and LL2). Given *Salmonella*'s influence on β3-adrenoceptor protein expression, we sought to identify the signaling pathway through which *Salmonella* mediates its effects on β3-adrenoceptor-mediated tumor metastasis. Previous studies have indicated that the protein kinase B (AKT) / mammalian target of rapamycin (mTOR) signaling pathway can promote many protein syntheses 12, 14. As expected, we observed a significant decrease in the elevated phosphorylation levels of AKT, mTOR, and p70S6K in B16F10 and LL2 cells upon treatment (Fig. 3 c and d). Furthermore, in a dose-dependent manner, *Salmonella* treatment led to a decrease in the phosphorylation of AKT, mTOR, and p70S6K, thereby indicating downregulation of the AKT/mTOR/p70S6K/β3-adrenoceptor pathway by *Salmonella* treatment in tumor cells (Fig. 3). These findings strongly suggest that the reduction in β3-adrenoceptor expression in tumor cells caused by *Salmonella* is associated with inhibiting the AKT/mTOR/p70S6K pathway. This sheds light on the potential mechanisms by which *Salmonella* exerts its anti-metastatic effects and provides valuable insights for further understanding its therapeutic potential in tumor treatment.

### 3.4. *Salmonella*-mediated reduction of β3-adrenoceptor expression was achieved by inhibiting the AKT signaling pathway

**Figure 4:**
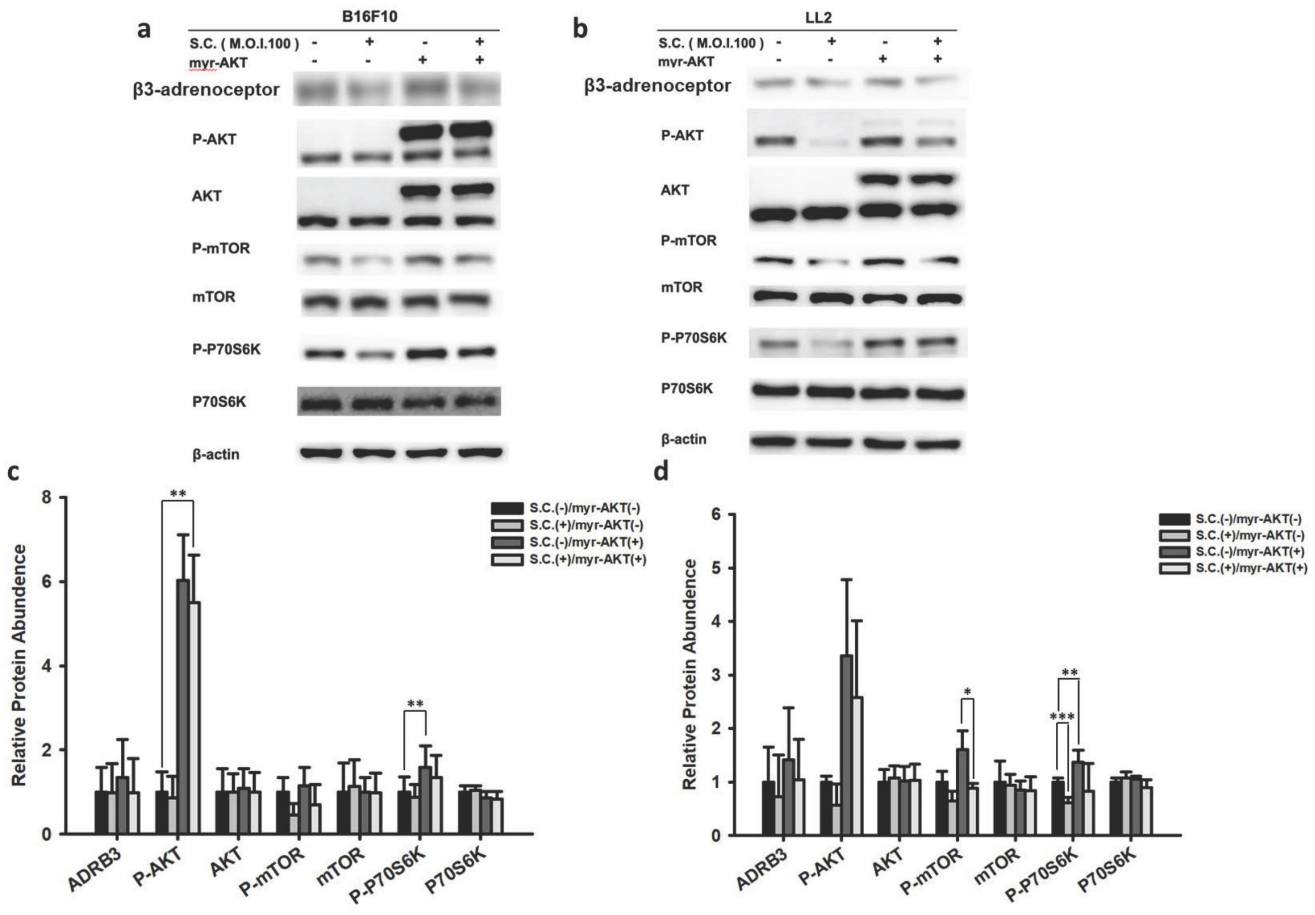
The AKT signaling pathways have participated in *Salmonella* (S.C.)-mediated β3-adrenoceptor expression. The B16F10 (a) and LL2 (b) cells were transfected with an active AKT plasmid. The cells were treated with *Salmonella* (MOI = 100) for 1.5 h after 16 h. The various protein expressions in B16F10 and LL2 cells were measured. The immunoblotting assay was repeated three times with similar results. The AKT/mTOR/p70S6K phosphorylation expression levels following *Salmonella* treatment in (a) B16F10 and (b) LL2 cells. Quantified band intensities normalized to β-Actin. (n = 3, mean ± SD. * p < 0.05; ** p < 0.01; *** p < 0.001).

**Figure 5:**
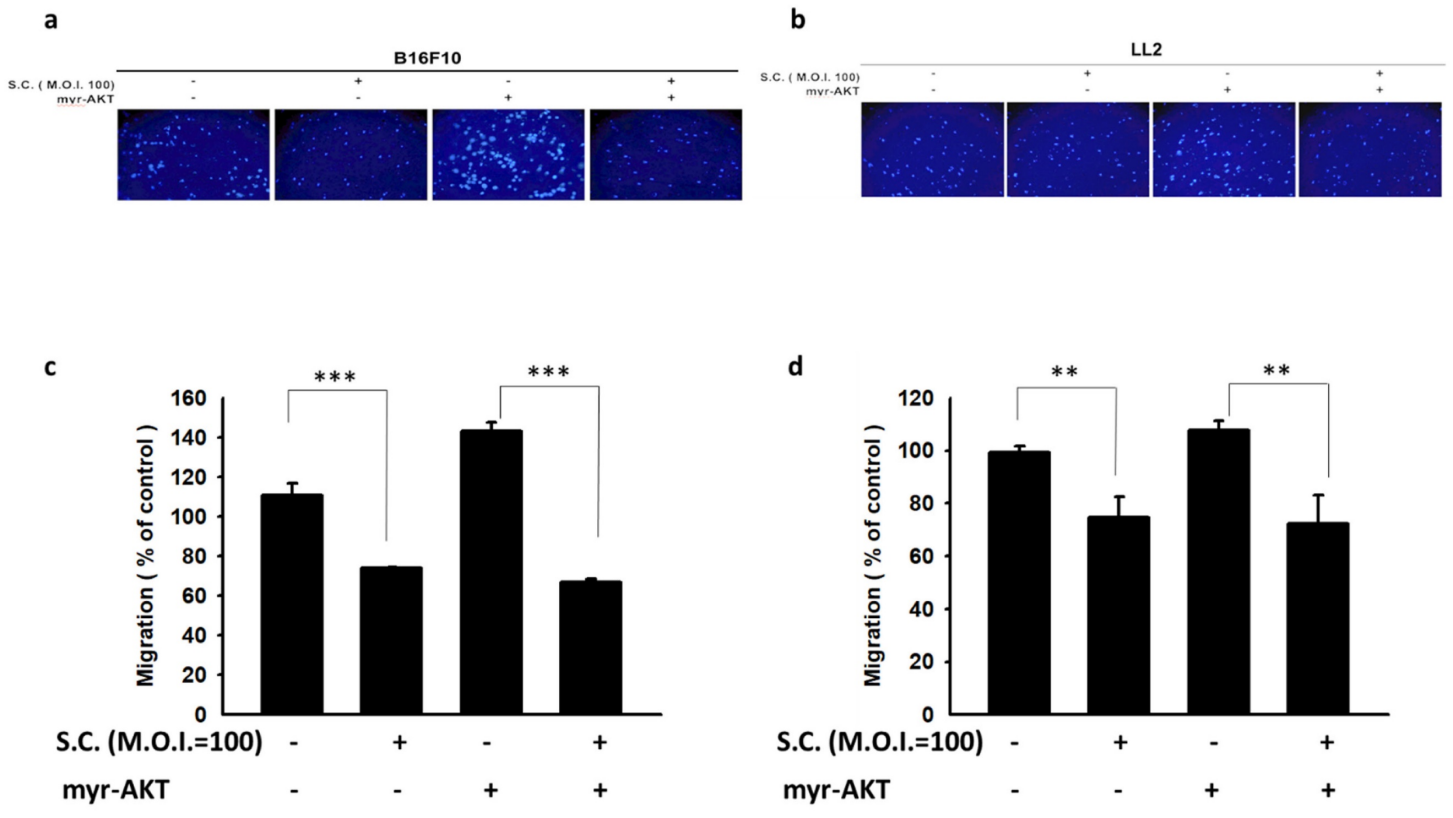
A Transwell assay showed that the AKT signaling pathways participated in the *Salmonella* (S.C.)-mediated inhibition of tumor cell migration. The B16F10 (a) and LL2 (b) cells were transfected with active AKT plasmids. After 16 h, the B16F10 and LL2 cells were placed on the upper layer of Tranwell and then infected with *Salmonella* (M.O.I. = 100) for 1.5 h. After 24 h, the bottom layer of cells was stained with 4',6-diamidino-2-phenylindole (DAPI) and counted under a fluorescence microscope in (c) B16F10 cells and (d) LL2 cells (n = 6, mean ± SD. ** p < 0.01; *** p < 0.001).

In this study, we observed that* Salmonella* reduced β3-adrenoceptor expression in tumor cells by decreasing AKT phosphorylation. To further investigate the role of the AKT/mTOR/p70S6K signaling pathway in this process, we conducted experiments involving the transfection of constitutively active AKT plasmids. When constitutively functional AKT plasmids were transfected into B16F10 (Fig. 4 a) and LL2 (Fig. 4 b) cells, the suppressive effect of *Salmonella* on the AKT/mTOR/p70S6K signaling pathway was reversed. The decrease in β3-adrenoceptor expression by Salmonella treatment is reversed by the transfection of active AKT plasmid However, this phenomenon was reversed after transfecting constitutively active AKT plasmids in the two tumor cell types (Fig. 4 c and d). We further utilized the Transwell assay to assess AKT's role in tumor cell migration. As shown in Fig. 5, *Salmonella* significantly reduced the movement of both B16F10 and LL2 cells. The capacity of *Salmonella* to hinder cell migration was significantly reduced. Indeed, there is no obvious difference that can be seen from Fig 5c and Fig. 5d in the two groups, both infected with *Salmonella*, with and without transfecting constitutively active AKT plasmids Depending on our experiment, *Salmonella* significantly inhibited the AKT-induced tumor cell migration.

These findings indicate that *Salmonella*'s ability to decrease β3-adrenoceptor expression and inhibit tumor cell migration is associated with its influence on the AKT/mTOR/p70S6K signaling pathway. The introduction of constitutively active AKT plasmids reversed these effects, emphasizing the critical role of AKT in mediating *Salmonella*'s anti-metastatic actions. Based on our findings, the downregulation of AKT is essential for *Salmonella*-induced reduction in β3-adrenoceptor expression and tumor cell migration.

### 3.5. *Salmonella*-mediated reduction of tumor cell migration *in vivo*

**Figure 6:**
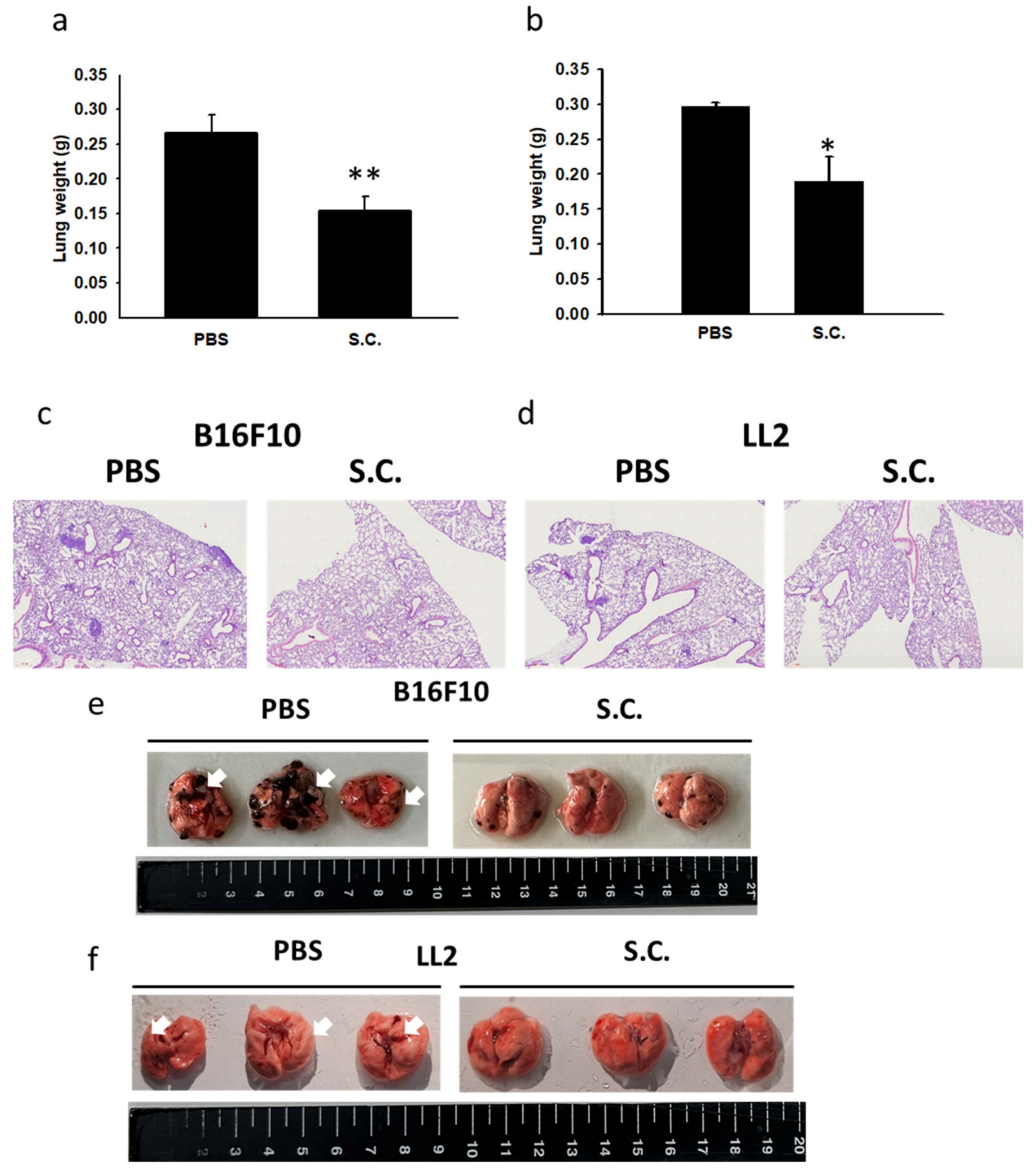

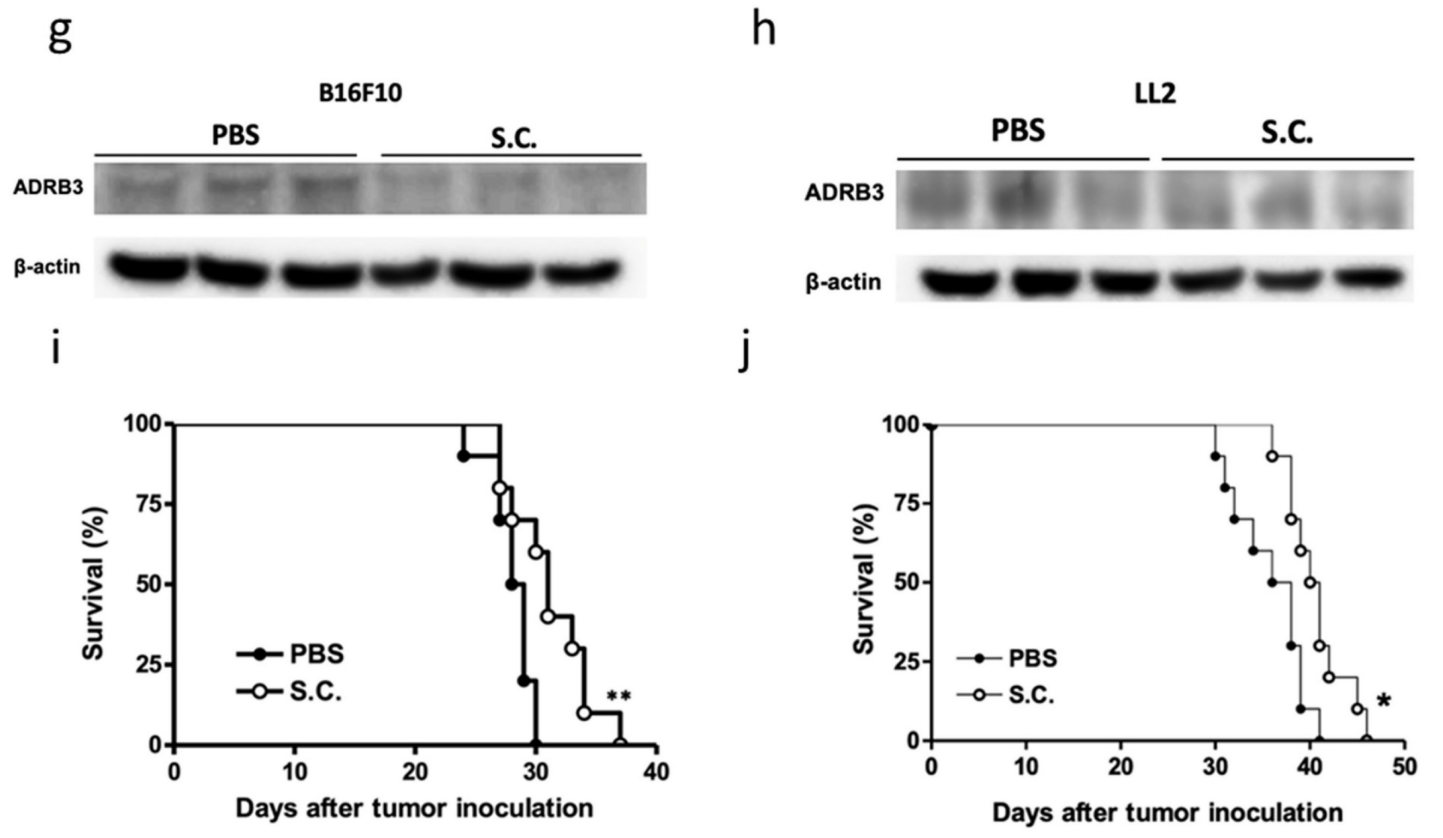
The expression of β3-adrenoceptor was reduced after *Salmonella* (S.C.) treatment *in vivo*. Mice were injected with tumor cells (10^5^) admixed with or without *Salmonella* (S.C.) (M.O.I. = 100) for 1.5 h via the tail vein. On Day 20, the mice were sacrificed. Mice were sacrificed, and the B16F10 (a) and LL2 (b) lung tumor weight was measured (n = 3). Histological lung tissue sections showing metastatic pulmonary tumor nodules were observed post-intravenous injection of B16F10 (c) and LL2 (d) cells. Effect of PBS or *Salmonella* o on lung tumor nodules in (e) B16F10 and (f) LL2 tumor models The B16F10 (g) and LL2 (h) in lung tissue protein levels of β3-adrenoceptor were measured-expression levels following *Salmonella* treatment in B16F10 and LL2 cells. Kaplan-Meier survival curves of mice bearing *Salmonella*-treated B16F10 (i) and LL2 (j) tumors are shown (n = 10, data are expressed as mean ± SD. * p < 0.05; ** p < 0.01).

Building upon our previous study, where we established mice models injected with tumor cells admixed with metastatic inducers or metastatic inhibitors to evaluate the activity of anti-metastatic molecules 6, we sought to determine whether *Salmonella* could inhibit metastasis *in vivo*. For this purpose, tumor cells pre-incubated with *Salmonella* were compared to those without *Salmonella* pre-treatment and injected into mice via the tail vein. Following *Salmonella* treatment, the mice were sacrificed, and the weight of lung tumors was measured. Remarkably, the lung tumors in *Salmonella*-treated mice exhibited a significantly lower weight than in the PBS control group, as shown in Fig 6 a and b. Moreover, the histological examinations of lung tissue sections revealed more tumor nodules in the mice that received B16F10 and LL2 cells alone (Fig.6 c and d). In contrast, those injected with cells admixed with *Salmonella* exhibited fewer tumor nodules (Fig 6 e and f). In addition, Western blotting analysis revealed reduced expression of β3-adrenoceptor in *Salmonella*-treated B16F10 and LL2 tumor-bearing mice (Fig. 6 g and h). The survival of the tumor-bearing mice treated with *Salmonella* was significantly enhanced compared to those treated with PBS in two tumor models (Fig. 5 i and j). These results provide compelling evidence that *Salmonella* indeed influences tumor metastasis* in vivo*. The findings suggest that *Salmonella* may hold potential as an effective agent in inhibiting tumor metastasis and warrant further investigation to validate its therapeutic applications in combating metastatic tumors.

## 4. Discussion

*Salmonella* offers numerous advantages as a potential treatment for tumors, encompassing tumor-targeting capabilities, immunostimulation, and cost-effectiveness 10. These attributes make *Salmonella* an attractive candidate for further exploration and development in tumor therapy. Understanding the mechanisms behind attenuated *Salmonella*'s targeting and preferential accumulation within tumors relative to normal tissues is an active area of research and remains incompletely understood 17. Tumors have a unique microenvironment that differs from normal tissues. Factors such as hypoxia (low oxygen levels), acidic pH, and increased vascular permeability can create a favorable niche for *Salmonella* to target and replicate within tumor tissues 18. A hallmark of many solid tumors is the hypoxic microenvironment. Hypoxia is also associated with a more malignant phenotype, affecting genomic stability, apoptosis, angiogenesis, and metastasis. The β3-adrenoceptor is significantly upregulated under hypoxic conditions 19. Previously, we showed that *Salmonella* could reduce hypoxia-inducible factor-1 α (HIF-1α) expression and may improve the hypoxic condition in the tumor microenvironment and increase the radiation or chemotherapy effects 20-22. *Salmonella* significantly inhibited tumor growth* in vivo*, and immunohistochemical studies of the tumors revealed decreased intratumoral microvessel density 20. Some studies reported reduced tumor volume and vasculature using β3-adrenoceptor blockades 23. *Salmonella* possesses similar capabilities to β3-adrenoceptor inhibitors.

The β3-adrenoceptors play a role in immunosuppression. Regulatory T cells (Tregs) are a subset of T cells that help maintain immune tolerance and prevent excessive immune responses. Activation of β3-adrenoceptors has been shown to modulate Treg function, potentially influencing immune tolerance 24.* Salmonella* has been studied for its potential as an immunostimulatory agent 14, 22. *Salmonella* can elicit an immune response against tumor cells when injected into the tumor or systemically administered 25. This approach is known as bacterial-based cancer immunotherapy. *Salmonella* can impact the tumor microenvironment, promoting a shift from an immunosuppressive to an immunostimulatory environment, making it more conducive for an effective immune response against the tumor. Based on our previous results, we found that *Salmonella* can accumulate in tumors after intraperitoneal injection 7.* Salmonella* can accumulate in tumors for over a month 11. *Salmonella* is not latent in tumor cells but accumulates in the tumor environment. Inhibition of tumor cell movement and metastasis through the interaction of* Salmonella* and the tumor microenvironment 5.

Lipolysis plays a critical role in tumor metabolism by providing fatty acids for energy production and biosynthesis. Tumors are adaptable and can utilize various substrates for energy, including lipids. Besides providing energy, the products of lipolysis can be used for the synthesis of new cellular membranes, signaling molecules, and other essential components for rapidly proliferating tumor cells. This is where lipolysis becomes essential. The β3-adrenoceptor plays an essential role in lipolysis by triggering a cascade of intracellular events that lead to the breakdown of fats 26. Activation of β3-adrenoceptors is associated with increased energy expenditure. This is because the breakdown of fats releases fatty acids that can be used as an energy source by various tissues in the body. *Salmonella* reduced the expression of β3-adrenoceptors in the tumor. In the future, we can explore whether *Salmonella* inhibits tumor growth by inhibiting tumor lipid metabolism.

*Salmonella* treatment targets β3-adrenoceptor and AKT/mTOR pathways in tumor growth and progression. It can be more effective and have fewer side effects than traditional therapy.* Salmonella* can stimulate the immune system to recognize and attack tumor cells, potentially preventing the spread of tumor cells to other organs. Further investigation is essential to comprehensively understand these inhibitory effects' underlying mechanisms and implications. This would entail conducting additional research, including preclinical and clinical studies, to validate the efficacy and safety of *Salmonella*-based therapies for tumor treatment. Such studies are crucial in advancing the potential use of *Salmonella* as a therapeutic approach to combat tumor metastasis, ensuring its viability as a safe and effective treatment option.

## Supplementary Material

Supplementary table 1.
