# Supplementary material for: Salmonella-induced inhibition of β3-adrenoceptor expression in tumors and reduces tumor metastasis
Source: J Cancer. 2024 Jan 1;15(5):1203–12. doi: 10.7150/jca.92024 (PMC10861817; doi:10.7150/jca.92024)
Supplement: Supplementary file 1 — Supplementary table 1. [file jcav15p1203s1.pdf]

Table S1. Antibodies Used for Western Blotting.

Antibody	TargetSize (kDa)	Host	Dilutions	Catalog No.	Company
β-Actin	43	Mouse	1:5000	A5441	Sigma-Aldrich, St. Louis, MO, USA
Phospho-Akt	~63	Mouse	1:500	sc-81433	Santa Cruz Biotechnology, CA, USA
Akt	~63	Rabbit	1:1000	sc-8312	Santa Cruz Biotechnology, CA, USA
Phospho-mTOR	289	Rabbit	1:1000	2983	Cell Signaling Technology, MA, USA
mTOR	289	Rabbit	1:1000	2974	Cell Signaling Technology, MA, USA
B3-adrenoceptor	44	Rabbit	1:1000	GTX54925	GeneTex, CA, USA
Phospho-p70S6K	70	Rabbit	1:1000	9205	Cell Signaling Technology, MA, USA
p-70S6K	70	Rabbit	1:1000	9202	Cell Signaling Technology, MA, USA
